# Prevalence and Clinical Characteristics of Allergic Fungal Rhinosinusitis: A Retrospective Analysis From Two Health Centers in Kuwait

**DOI:** 10.7759/cureus.83864

**Published:** 2025-05-10

**Authors:** Lulwah Al Saidan, Jafar Hayat, Mariam M Sarkhouh, Marwan Al-Qunaee

**Affiliations:** 1 Otolaryngology-Head and Neck Surgery, Kuwait Institute for Medical Specialization (KIMS), Kuwait City, KWT; 2 Surgery, Ministry of Health, Kuwait City, KWT; 3 Otolaryngology, Zain Hospital, Kuwait City, KWT

**Keywords:** functional endoscopic sinus surgery (fess), fungal sinusitis, kuwait, polyposis, sinusitis

## Abstract

Introduction

Allergic fungal sinusitis (AFS) is a noninvasive form of chronic rhinosinusitis (CRS) driven by an allergic response to fungal organisms, primarily affecting atopic individuals. It represents a clinically significant yet frequently underrecognized condition. AFS commonly presents with nasal polyps, and their co-occurrence can aid early diagnosis. Understanding this relationship is essential, as delayed recognition may lead to recurrent symptoms and the need for more aggressive intervention.

Methods

A retrospective cohort study was conducted on 110 adult patients with nasal polyposis who underwent functional endoscopic sinus surgery (FESS) between 2013 and 2019 at Zain Hospital and Jaber Al-Ahmad Al-Sabah Hospital in Kuwait. Patients were selected using consecutive sampling from operative records during the study period. The inclusion criteria encompassed adult patients of any age, gender, or nationality with clinically and radiologically confirmed nasal polyposis treated operatively at either center. The exclusion criteria included patients without nasal polyps, incomplete or missing medical records, sinonasal malignancies, non-inflammatory pathologies, or significant immunocompromising conditions unless stable at the time of surgery. Patients operated on outside the study centers were also excluded. Allergic fungal rhinosinusitis (AFRS) diagnosis was established through allergic mucin gram staining, computed tomography (CT) imaging, and histopathological confirmation. Primary outcome assessed AFRS prevalence; secondary outcomes analyzed recurrence patterns in relation to Lund-Mackay (LM) scores, fungal status, age, and gender.

Results

As per the histopathological findings for fungal sinusitis among the patients with nasal polyposis, 22/110 (20%) patients were positive for AFRS. An overall recurrence rate of 37.27% (41/110 patients) for patients with nasal polyps was found alongside a recurrence rate of 27.27% (6/22) for patients with AFRS. Recurrence rates were stratified according to Lund-Mackay (LM) scores of ≤12 and >12; results showed that recurrence rates for patients with LM scores of ≤12 were 39.1% (9/23) and 31.5% (23/73) for those with LM scores of >12. LM scores were not a significant factor in the recurrence rate of fungal sinusitis (χ²(1) = 0.03 and p = 0.86).

Conclusion

Identifying allergic fungal rhinosinusitis (AFRS) in patients with nasal polyps is critical for guiding appropriate management and improving outcomes. This study found a statistically significant association between gender and AFRS prevalence. Further research is needed to validate this correlation and to explore underlying mechanisms. Ongoing prevalence studies are also essential for understanding epidemiological trends and informing public health strategies.

## Introduction

Nasal polyps are defined as macroscopic edematous lesions originating from the mucosa of the nasal cavity or the sinuses. Inflammatory reactions have been linked with nasal polyp formation; however, the exact cause is still controversial. The prevalence of nasal polyps is approximately 1%-4% of the general population [1]. Eighty-five percent of nasal polyps are infiltrated by eosinophils marked by interleukin-5 (IL-5) [2]. The symptoms of the condition include anosmia, nasal obstruction, watery rhinorrhea, and recurrent infections of the nose and sinuses [3]. Chronic rhinosinusitis (CRS) is defined as a group of sinonasal disorders that are characterized by an inflammation of a minimum of 12 weeks in consecutive duration. CRS in recent definitions has been subclassified as CRS with nasal polyposis (CRSwNP), CRS without nasal polyposis (CRSsNP), and allergic fungal rhinosinusitis (AFRS) [4]. Symptoms usually present bilaterally, and it is important to rule out malignancy and other conditions in case of unilateral presentations [2]. The recurrence of nasal polyposis can reach up to 40% post-polypectomies [5]. Nasal polyps are linked with many conditions such as asthma, cystic fibrosis, and bronchiectasis. Samter's triad refers to the coexistence of nasal polyps, asthma, and aspirin sensitivity in a single patient [6]. Only 7% of patients with asthma have nasal polyps, while one-third of nasal polyposis patients are asthmatics, indicating a correlation between the two [5].

Fungal sinusitis is a common presentation in patients who suffer from nasal polyposis [7]. While typically considered benign, it can pose significant risks in immunocompromised patients, necessitating close monitoring and both medical and, when indicated, surgical management [8]. Allergic fungal sinusitis (AFS) can be extremely problematic as it has a high recurrence rate post surgical intervention and is characterized by nasal polyposis, the production of characteristic thick eosinophilic mucin, and aggressive, expansile changes of involved sinus cavities [9]. Type 1 hypersensitivity with immunoglobulin E (IgE) is pathologically associated with allergic fungal sinusitis. The condition is closely associated with asthma, atopy, and rhinitis [10]. In one article, Manning and Holman reported that almost 50% of fungal sinusitis patients have asthma in conjunction with their condition [11]. Nasal polyps are slightly more common in men compared to women, with a 2:1 ratio [12]. Allergic fungal sinusitis has no gender predominance [3,13]. As per the available literature, the incidence of fungal disease is higher in countries with high humidity [3]. The significance of conducting this study in Kuwait lies in its aim to assess the prevalence of allergic fungal sinusitis among patients with nasal polyposis in a region characterized by high humidity.

## Materials and methods

Study design

To study the prevalence of allergic fungal sinusitis (AFS) in patients with nasal polyposis, we conducted a retrospective cohort study. Between 2019 and 2023, 110 patients diagnosed with nasal polyposis who underwent functional endoscopic sinus surgery (FESS) at two otolaryngology departments in Kuwait were analyzed following clinical and radiological evaluation.

Correlations were drawn between variables such as age, gender, recurrence, and Lund-Mackay (LM) scores.

Perioperative management

All patients underwent preoperative sinus computed tomography (CT) scans, and intraoperative biopsy samples were collected for histopathological assessment. The diagnosis of allergic fungal rhinosinusitis (AFRS) was confirmed based on the presence of eosinophilic mucin containing fungal hyphae, identified through hematoxylin and eosin (H&E) staining as the primary method. Potassium hydroxide (KOH) mounts were used when fungal infection was suspected, and standard fungal cultures were performed for species identification when appropriate.

Postoperatively, all patients received functional endoscopic sinus surgery (FESS), followed by adjunctive therapies tailored to individual cases. As part of the postoperative management, all patients were prescribed topical nasal corticosteroids and saline irrigations for a minimum of two months to reduce inflammation and prevent recurrence.

Systemic antifungal therapy, typically itraconazole 100 mg twice daily, was prescribed for 3-6 months, depending on the disease severity and in accordance with current treatment guidelines. Intranasal steroid sprays were initiated postoperatively, following FESS, to further control inflammation and prevent recurrence, continuing for at least two months.

Follow-up visits were scheduled at two weeks, one month, three months, six months, and 12 months, during which recurrence was assessed based on symptoms, nasal endoscopy, and CT scans.

Inclusion criteria

Adult patients of any gender, age, or nationality with nasal polyposis who underwent functional endoscopic sinus surgery (FESS) in the past 10 years in the two otolaryngology-head and neck departments of Zain Hospital and Jaber Al-Ahmad Al-Sabah Hospital were included in the study.

Exclusion criteria

Patients who underwent FESS without a diagnosis of nasal polyposis (regardless of fungal etiology), patients with incomplete or missing medical records, patients with concurrent sinonasal malignancies or other non-inflammatory sinonasal pathologies, and patients with systemic conditions that significantly alter immune response or healing (e.g., HIV/AIDS and chemotherapy recipients) unless their condition was stable and well-documented at the time of surgery were excluded from the study. We had also excluded all patients who were managed operatively outside of these two centers.

Data collection and histopathology and imaging protocols

Patient data were retrospectively collected from electronic medical records, operative logs, and pathology databases at two tertiary otolaryngology centers in Kuwait, Zain Hospital and Jaber Al-Ahmad Al-Sabah Hospital, covering the period from January 2019 to August 2023. Data extraction was performed by two co-authors (JH and MS) using a standardized data collection form to enhance consistency across variables. Blinding was not feasible due to the retrospective study design. Some variability in data completeness was unavoidable.

Histopathological analysis was performed at institutional pathology departments using standard hematoxylin and eosin (H&E) staining. Special fungal stains such as Grocott-Gömöri methenamine silver (GMS) were applied when fungal infection was suspected intraoperatively. Slides were reviewed by attending pathologists at the respective centers. Imaging was conducted using multi-slice CT scanners; however, details such as slice thickness and specific criteria for hyperattenuation were not uniformly recorded.

The following variables were recorded: demographic data: age, gender, and nationality; clinical history: presenting symptoms, a history of allergic rhinitis or asthma, and previous sinus surgeries; radiological findings: preoperative Lund-Mackay CT scores; intraoperative findings: the presence of fungal debris, the extent of polyposis, and mucin characteristics; histopathological results: the presence of eosinophilic mucin, fungal hyphae, fungal culture results, and KOH mount findings; treatment details: surgical intervention, postoperative steroid sprays (duration and type), and the use of antifungal therapy (based on culture sensitivity and clinical indication); and follow-up outcomes: the recurrence of symptoms, need for revision surgery, and radiologic follow-up findings. All patients had a minimum of 12 months of clinical follow-up, unless lost to follow-up. Follow-up was conducted in a clinic setting using standard devices. Recurrence was defined as the return of symptoms and/or radiological or endoscopic findings consistent with disease within 12 months, including the need for revision surgery.

Data handling

Missing data were an inherent limitation due to the retrospective design of the study. Patients with incomplete data were excluded from specific subgroup analyses but remained included in the overall demographic and prevalence assessments. For instance, of the 110 patients, 96 had complete imaging data available for Lund-Mackay scoring and were included in the radiologic subgroup analyses.

Ethical approval statement

This study was approved by the Kuwait Ministry of Health's Research Ethics Review Board. All procedures were performed in accordance with ethical standards set by the Declaration of Helsinki, and patient confidentiality was maintained throughout the study.

Statistical analysis

The dataset of 110 patients was analyzed using IBM SPSS® Statistics (IBM Corp., Armonk, NY). Variables were stratified based on demographic and clinical characteristics, and comparisons were made to explore potential associations. Given the retrospective and exploratory nature of the study, descriptive statistics were used to summarize the data. A chi-square test was employed for categorical variables. For continuous variables, independent samples t-tests were employed when assumptions of normality were met.

Chi-square tests were applied to all categorical data groups. Yates' correction was not used. A p-value of <0.05 was considered statistically significant. The primary variables assessed included recurrence rates stratified by Lund-Mackay (LM) scores in fungal versus non-fungal cases, the prevalence of fungal sinusitis by gender and age, and recurrence rates of nasal polyposis by gender and age.

## Results

The medical records of 110 patients were involved in this retrospective study. The patients discussed were within the age ranges of 7-78, with a mean age of 36.7 years and a standard deviation of ±13.985 years. There were 40 female patients (36.3%) and 70 male patients (63.6%). CT scores using the Lund-Mackay grading system were available for 96 patients.

As per available histopathology results, the incidence of AFS for the patients who presented for FESS for nasal polyposis was 20% (22/110 patients). The remainder of the patients yielded negative findings for fungus in the histopathological samples sent out.

**Figure 1:**
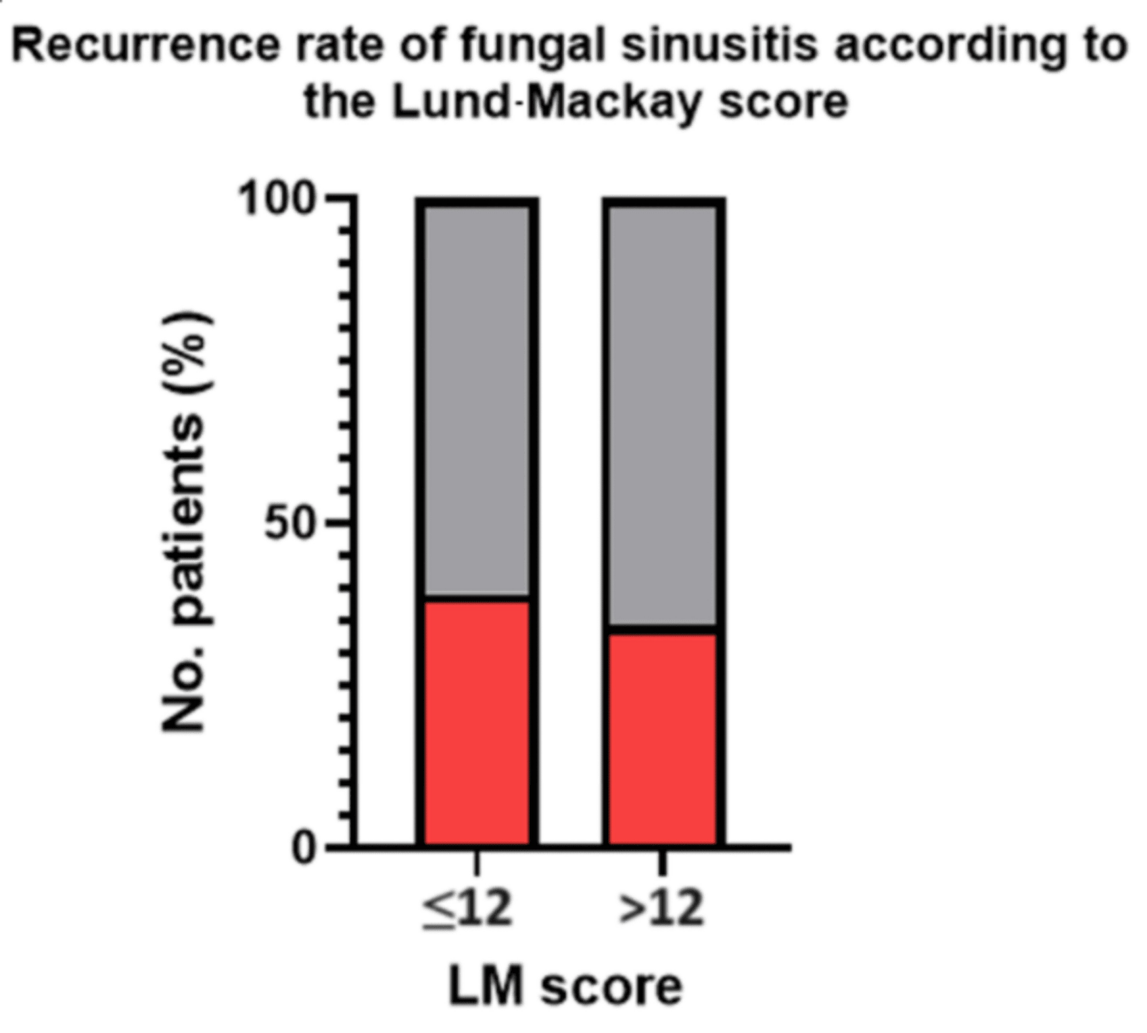
Recurrence of Nasal Polyposis According to Lund-Mackay (LM) Scores

The overall recurrence rate of non-fungal nasal polyposis in the dataset yielded a result of 39.77% (35/88 patients). There was a 27.27 (6/22) recurrence rate for patients who were diagnosed with AFS. The recurrence rate of fungal sinusitis according to the Lund-Mackay (LM) score was divided into two categories: those with a score of more than 12 and a score of less than or equal to 12. The recurrence rate of an LM score of ≤12 was 39.1% (9/23) and >12 was 31.5% (23/73). LM score was not a significant factor for the recurrence rate of fungal sinusitis (χ²(1) = 0.18 and p = 0.669). Table 1 and Figure 1 depict these findings.

**Table 1:** Recurrence of Nasal Polyposis According to Lund-Mackay Scores Comparison: Lund-Mackay versus recurrence test used: chi-square test; p < 0.05 is deemed statistically significant

Variable	≤12 (n = 23)	>12 (n = 73)	χ²	P-value
Recurrence: yes	9 (39.13%)	25 (34.25%)	0.18	0.669
Recurrence: no	14 (60.87%)	48 (65.75%)		

**Figure 2:**
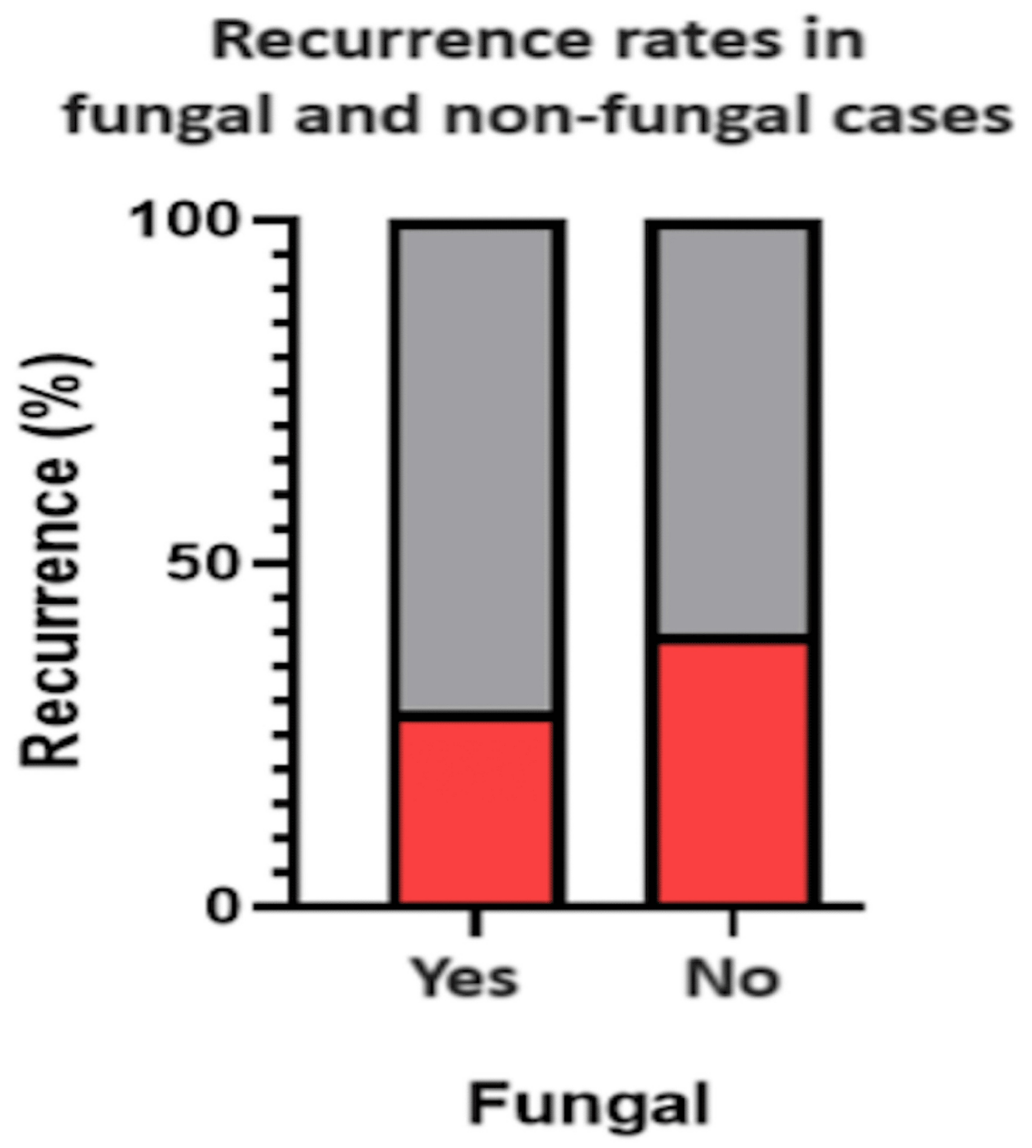
Recurrence Rates in Fungal and Non-fungal Cases

**Figure 3:**
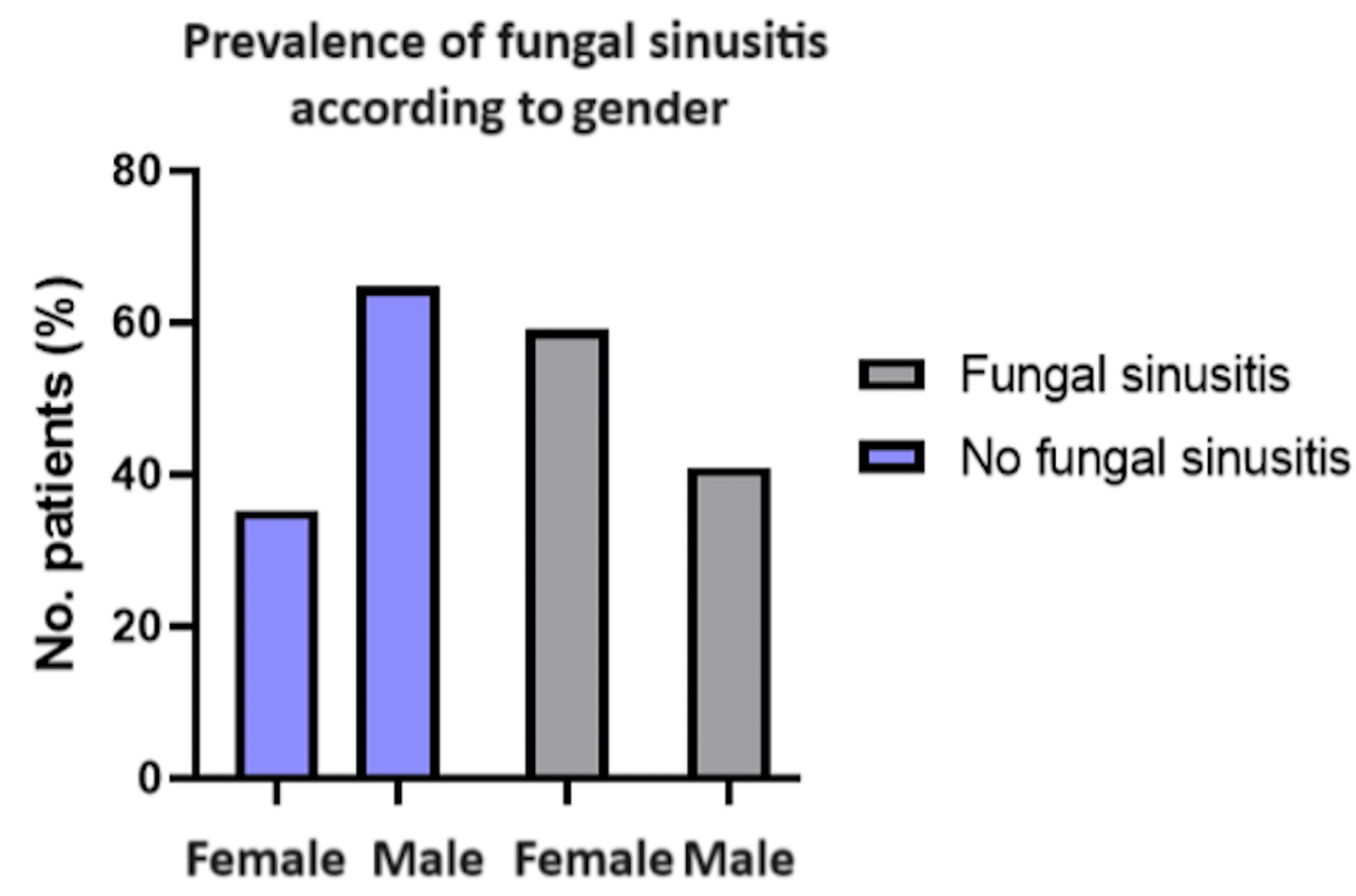
Prevalence of Fungal Sinusitis According to Gender

The recurrence rate for nasal polyps was 37.27% (41/110 patients) within a one-year follow-up. There was no significant association between fungal sinusitis and recurrence in comparison to non-fungal cases (χ²(1, N = 110) = 1.18 and p = 0.278), as seen in Table 2 and Figure 2. There was a statistically significant association between gender and fungal sinusitis (χ²(1, N = 110) = 4.18 and p = 0.041). This is seen in Table 3 and Figure 3.

**Table 2:** Recurrence Rates in Fungal and Non-fungal Cases Comparison: fungal versus non-fungal recurrence test used: chi-square test; p < 0.05 is deemed statistically significant

Variable	Fungal (n = 22)	Non-fungal (n= 88)	χ²	P-value
Recurrence: yes	6 (27.27%)	35 (39.77%)	1.18	0.278
Recurrence: no	16 (72.73%)	53 (60.23%)		

**Table 3:** Prevalence of Fungal Sinusitis According to Gender Comparison: gender versus fungal sinusitis test used: chi-square test; p < 0.05 is deemed statistically significant

Gender	Fungal (n = 22)	Non-fungal (n = 88)	χ²	P-value
Male	9 (40.91%)	57 (64.77%)	4.18	0.041
Female	13 (59.09%)	31 (35.23%)		

**Figure 4:**
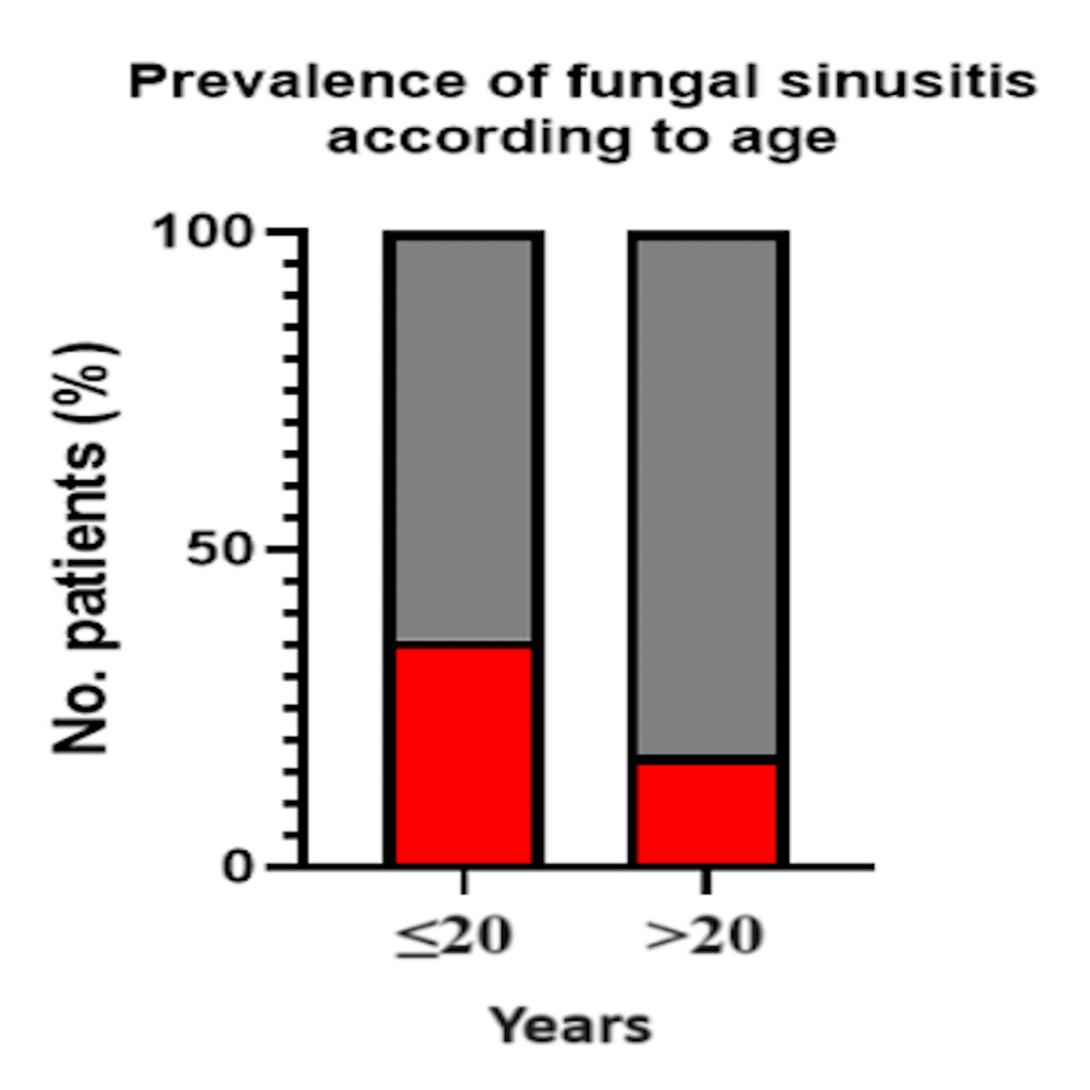
Prevalence of Fungal Sinusitis According to Age

**Figure 5:**
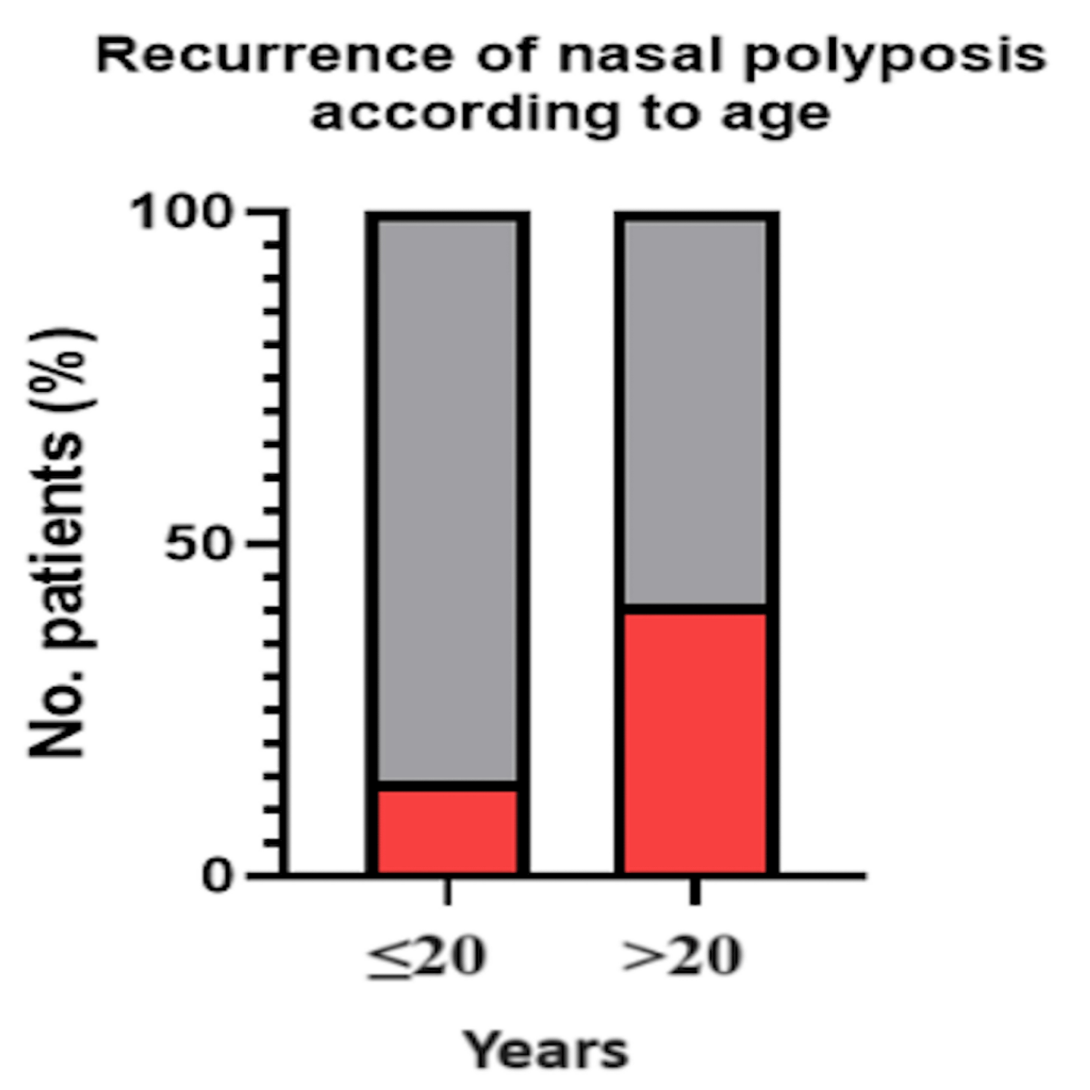
Recurrence of Nasal Polyposis According to Age

There was no statistically significant association between age group and fungal sinusitis (χ²(1, N = 110) = 2.48 and p = 0.116) (Table 4 and Figure 4), nor was there any significant association between age and the recurrence of nasal polyposis (χ²(1, N = 110) = 3.73 and p = 0.054) (Table 5 and Figure 5). This p-value however does represent a correlation.

**Table 4:** Prevalence of Fungal Sinusitis According to Age Comparison: age versus fungal sinusitis test used: chi-square test; p < 0.05 is deemed statistically significant

Age group	Fungal: yes	Fungal: no	χ²	P-value
≤20 (n = 14)	5 (35.71%)	9 (64.29%)	2.48	0.116
>20 (n = 96)	17 (17.71%)	79 (82.29%)		

**Table 5:** Recurrence of Nasal Polyposis by Age Group Chi-square test was used to compare nasal polyposis recurrence by age group. Statistical significance was considered at p < 0.05

Age group	Recurrence: yes	Recurrence: no	χ²	P-value
≤20 (n = 14)	2 (14.29%)	12 (85.71%)	3.73	0.054
>20 (n = 96)	39 (40.63%)	57 (59.38%)		

**Figure 6:**
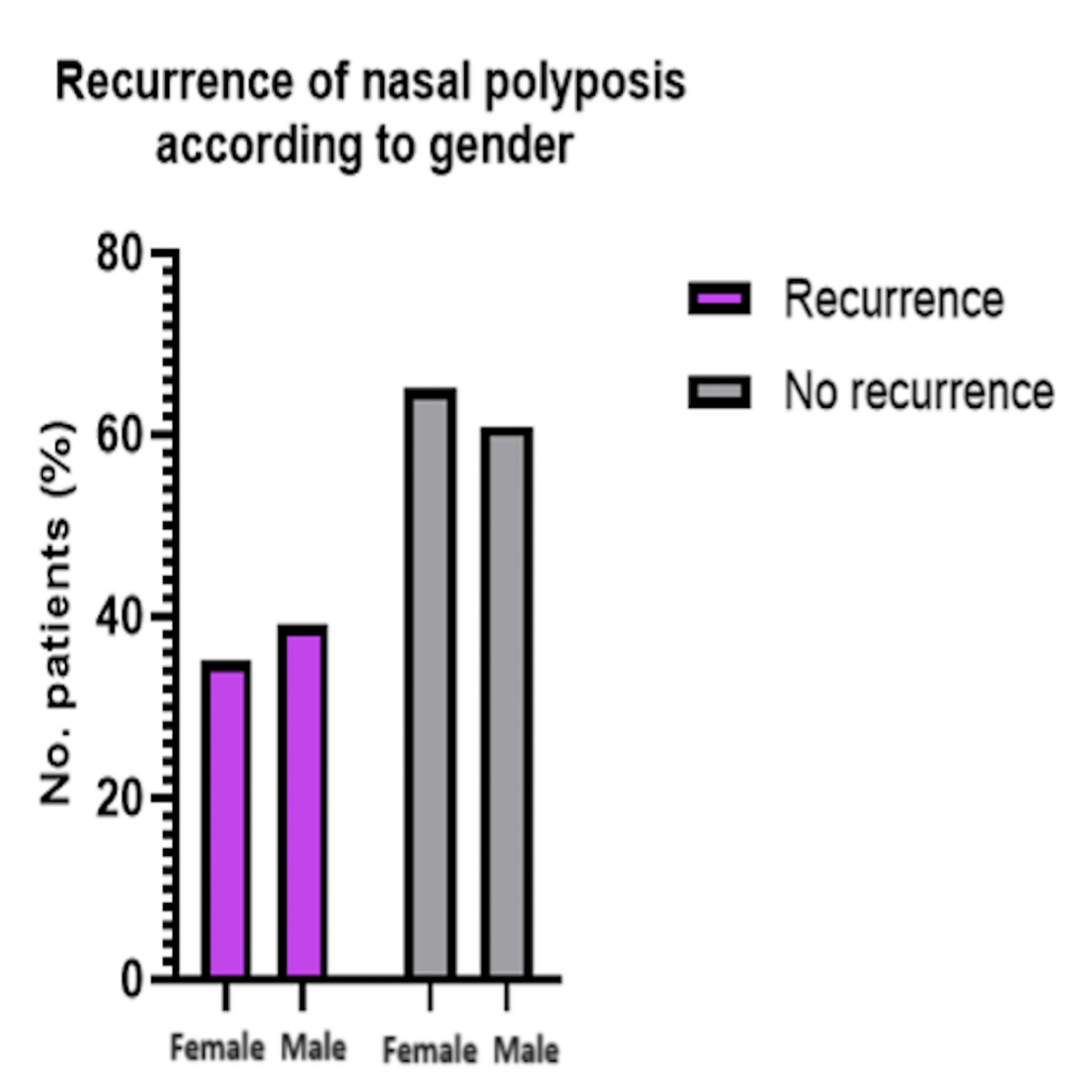
Recurrence of Nasal Polyposis According to Gender

Lastly, there was also no significant association between gender and the recurrence of nasal polyposis (χ²(1, N = 110) = 0.27 and p = 0.601). This can be seen in Table 6 and Figure 6.

**Table 6:** Recurrence of Nasal Polyposis According to Gender Comparison: gender versus recurrence test used: chi-square test; p < 0.05 is deemed statistically significant

Gender	Recurrence: yes	Recurrence: no	χ²	P-value
Male (n = 69)	27 (39.13%)	42 (60.87%)	0.27	0.601
Female (n = 41)	14 (34.15%)	27 (65.85%)		

## Discussion

The suspicion of allergic fungal rhinosinusitis (AFRS) typically arises in the outpatient setting, based on clinical history and findings from flexible nasal endoscopy. Sinus CT scans and occasionally MRI scans are also ordered, with the scans showing regions of hyperattenuation [14]. Definitive diagnosis however is achieved intraoperatively via histopathological findings. The standard finding reveals stainings of pyknotic and degranulated eosinophils surrounded by areas of lightly staining mucin sprinkled with Charcot-Leyden crystals [15].

The most widely adopted criteria to diagnose AFRS were published by Bent and Kuhn in 1994 [16]. Five major criteria were named. The criteria that were mentioned include there being a type 1 IgE-mediated hypersensitivity present, there being nasal polyposis, there being a characteristic computed tomography (CT) finding(s), there being eosinophilic mucus, and lastly there being a positive fungal smear [17]. In these criteria, patients must meet all the major criteria for diagnosis. Six minor criteria also exist, which include a diagnosis of asthma, unilateral disease being present, bony erosions being present, positive fungal cultures, Charcot-Leyden crystals found, and a raised serum eosinophilia level. However, the minor criteria serve to support the diagnosis and describe individual patients but are not used to make a diagnosis [18].

Literature suggests that AFRS is more prevalent in hot and humid climates [19]. Dhanani et al. (2021) discovered that 23.7% of patients with nasal polyposis had AFRS, from a sample size of 114, when retrospectively assessing prevalence in Karachi, Pakistan, a region with a similar climate to the data collected from Kuwait [20]. Hoyt et al. (2016) from Louisiana discussed that AFRS locally accounts for 5%-10% of chronic rhinosinusitis (CRS) cases. This difference in incidence is suggested to be due to geographic and diagnostic differences [19].

During surgery, which is the mainstay of the management of AFRS, preserving mucosal health for mucociliary function is essential. Surgical procedures should prioritize preserving mucosal integrity to ensure effective mucociliary clearance. Excessive tissue removal, such as turbinate resection, may compromise nasal function, such as sacrificing turbinates, risks impairing nasal function, reducing the sense of smell, and leading to complications such as "empty nose syndrome" [21]. A balanced surgical approach is critical to maintaining long-term physiological functionality. Thorough debridement, including the removal of bony partitions that may harbor fungal debris and mucin, is essential to reduce antigenic stimulation and the recurrence of the condition. Inadequate clearance can lead to inaccessible sinus areas where allergens persist, triggering ongoing inflammatory responses.

In addition to that, proper surgical marsupialization is vital for ensuring postoperative topical medications reach deeper sinus areas, such as retro-maxillary cells and the lateral recess of the sphenoid sinus. This approach minimizes the retention of fungal debris and allergic mucin, preventing recurrent inflammation.

Moreover, comprehensive surgical techniques, including fronto-sphenoethmoidectomy and wide maxillary antrostomy, are essential for clearing fungal mucin and improving sinus ventilation. Wide sinus openings facilitate better drainage and enhance the effectiveness of postoperative topical treatments [22].

The postoperative management of AFRS includes, but is not limited to, intranasal corticosteroids and nasal rinses, and in some instances, courses of systemic corticosteroids might be required [23].

Polyp recurrence is common after FESS, with the control of polyps up to 18 months found in approximately 60%-70% of patients [24]. Immunotherapeutics such as IL-4, IL-13, and IL-5 inhibitors provide promising results in improving CRSwNP patient outcomes. Evidence mounted over the last few years shows the effectiveness of biological therapy in patients with AFRS [25].

In regard to the strengths of this study, this study provides a comprehensive dataset from two tertiary centers in Kuwait, offering valuable insights into the prevalence and management of allergic fungal rhinosinusitis (AFRS) in the region. The inclusion of a relatively large cohort of 110 patients over a four-year period strengthens the validity of the findings, making it a significant early contribution to local epidemiological data. By utilizing objective measures such as Lund-Mackay CT grading and histopathology (including fungal cultures and H&E staining), the study enhances diagnostic reliability. The regional focus addresses a gap in the literature related to AFRS in Middle Eastern populations, which is vital for informing healthcare strategies in similar climates.

This study is subject to several limitations inherent to its retrospective design, which limits its ability to control for biases related to data completeness, patient selection, and clinical documentation. One key limitation is the lack of standardized data collection between the primary and secondary centers, which may have introduced discrepancies in diagnostic protocols, particularly with respect to ordering and interpreting laboratory tests such as IgE levels and eosinophil counts. Variability in how these laboratory tests were ordered and processed across centers could have influenced the results, potentially leading to inconsistencies in diagnosing and managing AFRS. Additionally, the study was conducted at only two healthcare centers in Kuwait, limiting the generalizability of the findings to other regions or healthcare settings with different diagnostic practices and patient populations. The absence of long-term follow-up, with a maximum follow-up period of one year, restricts our understanding of AFRS recurrence and disease progression. Given the known recurrence rate of AFRS, a longer follow-up period would have provided more comprehensive insights into long-term outcomes. While the study utilized the Lund-Mackay scoring system for preoperative CT assessments, it was not explicitly stated whether all cases followed the Bent and Kuhn criteria for diagnosing AFRS perioperatively via a checklist, potentially leading to inconsistencies in case identification, a limitation that can be addressed in future studies. There is also a possibility of the underdiagnosis of AFRS in patients without histopathology or those lost to follow-up, which may have affected the overall study conclusions. Despite its limitations, this study offers valuable regional insights that could serve as a foundation for future research and clinical practice in the field.

## Conclusions

This study evaluated the prevalence of allergic fungal rhinosinusitis (AFRS) in patients with nasal polyposis in Kuwait. We found a significant association between AFRS and specific demographics and correlations with clinical characteristics. This emphasizes the importance of early detection and management. The study highlights the utility of Lund-Mackay scores and histopathology in identifying at-risk patients.

Given the regional focus, future research should aim to validate these findings in larger, more diverse populations, with longer follow-up to better understand recurrence and disease progression. Standardizing diagnostic protocols and improving data collection will be essential for advancing care in the region.
